# Incidence of laboratory-confirmed influenza and RSV and associated presenteeism and absenteeism among healthcare personnel, Israel, influenza seasons 2016 to 2019

**DOI:** 10.2807/1560-7917.ES.2024.29.31.2300580

**Published:** 2024-08-01

**Authors:** Eduardo Azziz-Baumgartner, Avital Hirsch, Young M Yoo, Alon Peretz, David Greenberg, Yonat Shemer Avni, Aharona Glatman-Freedman, Michal Mandelboim, Adam MacNeil, Emily T Martin, Gabriella Newes-Adeyi, Mark Thompson, Arnold S Monto, Ran D Balicer, Min Z Levine, Mark A Katz

**Affiliations:** 1 United States Centers for Disease Control and Prevention (CDC), Atlanta, United States; 2 Clalit Research Institute, Innovation Division, Clalit Health Services, Tel Aviv, Israel; 3 Rabin Medical Center, Clalit Health Services, Petah Tikva, Israel; 4 Soroka University Medical Center, Clalit Health Services, Beersheba, Israel; 5 Israel Center for Disease Control, Ramat Gan, Israel; 6 School of Public Health, Faculty of Medical and Health Sciences, Tel Aviv University, Tel Aviv, Israel; 7 Central Virology Laboratory, Ministry of Health, Tel-Hashomer, Israel; 8 School of Public Health, University of Michigan, Ann Arbor, Michigan, United States; 9 Abt Associates Inc., Atlanta, United States; 10 School of Public Health, Faculty of Health Sciences, Ben Gurion University of the Negev, Be'er Sheva, Israel

**Keywords:** flu, RSV, incidence, presenteeism, absenteeism, healthcare personnel, Israel

## Abstract

**Background:**

Healthcare personnel (HCP) are at high risk for respiratory infections through occupational exposure to respiratory viruses.

**Aim:**

We used data from a prospective influenza vaccine effectiveness study in HCP to quantify the incidence of acute respiratory infections (ARI) and their associated presenteeism and absenteeism.

**Methods:**

At the start and end of each season, HCP at two Israeli hospitals provided serum to screen for antibodies to influenza virus using the haemagglutination inhibition assay. During the season, active monitoring for the development of ARI symptoms was conducted twice a week by RT-PCR testing of nasal swabs for influenza and respiratory syncytial virus (RSV). Workplace presenteeism and absenteeism were documented. We calculated incidences of influenza- and RSV-associated ARI and applied sampling weights to make estimates representative of the source population.

**Results:**

The median age of 2,505 participating HCP was 41 years, and 70% were female. Incidence was 9.1 per 100 person-seasons (95% CI: 5.8–14.2) for RT-PCR-confirmed influenza and 2.5 per 100 person-seasons (95% CI: 0.9–7.1) for RSV illness. Each season, 18–23% of unvaccinated and influenza-negative HCP seroconverted. The incidence of seroconversion or RT-PCR-confirmed influenza was 27.5 per 100 person-seasons (95% CI: 17.8–42.5). Work during illness occurred in 92% (95% CI: 91–93) of ARI episodes, absence from work in 38% (95% CI: 36–40).

**Conclusion:**

Influenza virus and RSV infections and associated presenteeism and absenteeism were common among HCP. Improving vaccination uptake among HCP, infection control, and encouraging sick HCP to stay home are important strategies to reduce ARI incidence and decrease the risk of in-hospital transmission.

**Key public health message**

**What did you want to address in this study and why?**

Seasonal influenza vaccination is widely recommended for healthcare personnel (HCP), but little is known about the risk of respiratory infections like influenza and respiratory syncytial virus (RSV) among HCP and about how respiratory illnesses contribute to absenteeism (missing work) and presenteeism (working while ill) among HCP. We undertook a study to answer these questions to help inform mitigation measures and return-to-work policies for HCP.

**What have we learnt from this study?**

Respiratory virus infections were common among HCP in two Israeli hospitals. Unvaccinated HCP had 9.1 influenza episodes per 100 people (on average across the three influenza seasons) confirmed through testing of nasal swabs. That rate more than doubled when we added infections identified through blood tests for antibodies. Most HCP worked at least 1 day while sick and workplace absenteeism resulted in more than 23,000 hours of lost work.

**What are the implications of your findings for public health?**

We found high incidence of acute respiratory illness (ARI), influenza and RSV infections and workplace presenteeism and absenteeism among HCP. Improving influenza vaccination coverage, considering the introduction of RSV vaccines, optimising infection control measures, and encouraging sick HCP to stay home are important strategies to reduce the burden of ARI, optimise healthcare system resilience and reduce healthcare-associated transmission.

## Background

While it is useful to know the incidence of influenza and respiratory syncytial virus (RSV)-associated acute respiratory illnesses (ARI) when assessing the cost–benefit balance of vaccination, few countries have generated such findings for potential target groups such as healthcare personnel (HCP) [1]. Compared with other community-dwelling adults, HCP are disproportionally exposed to respiratory viruses through work and are often at higher risk for infections [2]. Workplace absenteeism, and associated costs of lost productivity among HCP, is also an important component of the financial burden to society and of potential utility when estimating costs and benefits of vaccination.

Better understanding the burden of influenza and RSV infections among HCP can inform prioritisation of public health resources, including influenza vaccination [3]. In addition, while vaccine acceptance is critical to the success of influenza vaccination campaigns [4,5], HCP are often reluctant to use or recommend vaccines because of uncertainty about their risk of infection [6]. Appreciation of influenza illness risk, for example, can motivate HCP to get vaccinated against influenza [7].

The incidence of influenza and RSV infection among children [8-10] and older adults [11,12], two populations prioritised for influenza vaccination, has been well quantified in Israel. While Israel also recommends seasonal influenza vaccines for HCP, little is known about HCP’s risk of influenza and illness impact on presenteeism and absenteeism [13]. We leveraged data from a prospective cohort study of influenza vaccine effectiveness among HCP in Israel [14] and quantified the incidence of all-cause ARI, influenza and RSV illnesses, as well as presenteeism and absenteeism associated with these illnesses.

## Methods

During the 2016/17, 2017/18, and 2018/19 influenza seasons, HCP at the Soroka and Rabin Medical Centers in Israel were enrolled as described previously [14]. Briefly, at the start of each season, HCP at both participating hospitals were enrolled in the study using a stratified random sampling approach across 36 strata of age, sex (binary: male or female) and occupation to improve representativeness. Following written informed consent, HCP completed an enrolment survey that included questions about sociodemographic and occupational characteristics. Data on pre-existing conditions and influenza vaccination were collected from electronic medical records and hospital employee vaccination registries. We considered HCP as vaccinated if they received their influenza vaccine at least 2 weeks before the peak of the influenza season. Participating HCP also provided serum specimens to screen for antibodies to influenza virus using the haemagglutination-inhibition (HI) assay at the start and end of each influenza season. Timing of each influenza season was determined by the first and last influenza virus infections detected in the cohort via RT-PCR: the epidemic period was epidemiological week W49 in 2016 through W11 in 2017 for 2016/17 season, W48 in 2017 through W12 in 2018 for the 2017/18 season, and W48 in 2018 through W10 in 2019 for the 2018/19 season.

During influenza seasons, enrolled HCP began active surveillance for development of ARI within the past 7 days [14]. Participants received twice-weekly short message service (SMS) surveillance messages to report any ARI symptoms. If they did not respond to two consecutive messages, they received reminder calls from study staff. They were also encouraged to contact study staff directly if they experienced ARI symptoms. If the HCP reported symptoms, they had nasal swab samples collected by self-collection or study staff [15,16], and samples were tested for influenza and RSV ribonucleic acid by RT-PCR at the Soroka Hospital Laboratory. Symptomatic HCP completed a confidential questionnaire about whether they worked while ill with any ARI symptom (defined as presenteeism) or missed work because of illness (defined as absenteeism); questionnaire data were compared and reconciled with each hospital’s human resources records. If HCP reported missed work, we recorded the number of hours missed, but the questionnaire did not capture the number of hours worked while ill. The HCP also self-rated their ability to do work activities during illness on a scale from 0 to 9, where 0 was inability to do any usual activity and 9 was ability to do all usual activities. This scale has previously been validated to evaluate the impact of acute respiratory illness on activities of daily living among HCP [17-19].

We defined ARI as an illness with at least one of the following symptoms: cough, feverishness (i.e. subjective or measured fever), rhinorrhoea or body aches, regardless of aetiology or severity [14]. Illness duration was determined as the number of days between onset of the first symptom and complete symptom resolution. We considered reported ARI as separate episodes if more than 14 days had passed between symptom resolution of the preceding ARI and symptoms onset of the subsequent episode. If a person had two different infections with the same virus (e.g. influenza A and influenza B or influenza A(H3) and influenza A(H1N1)) in the same season, these were treated as two separate illnesses. Unvaccinated HCP who did not meet the ARI case definition but demonstrated serological evidence of natural infection were classified as having asymptomatic infections. Serological evidence of influenza infection was defined as a ≥ 4-fold rise in HI antibody titres from pre- to post-season serum samples (i.e. seroconversion) and a ≥ 1:40 HI titre on the post-season sample. Serology samples were not systematically tested for RSV.

Cumulative incidence of ARI, influenza-associated ARI and RSV-associated ARI was calculated by dividing the number of illness episodes by the number of study participants per influenza season (i.e. the proportion of people who develop an ARI during an influenza season) and is expressed as person-season. During missed surveillance weeks, HCP were assumed to be without illness. In addition, we calculated cumulative incidence of ARI-associated presenteeism and absenteeism by dividing the number of presenteeism or absenteeism events during ARI episodes by the person-seasons. We applied sampling weights to generate incidence estimates representative of the source population (i.e. all HCP employed at Soroka or Rabin Medical Centers and members of Clalit Health Services).

To examine demographical or occupational [20] factors (e.g. differences in respiratory illness risk by ethnicity) associated with ARI, influenza-associated ARI and RSV-associated ARI incidence, we used Poisson regression to calculate relative risk. Generalised estimating equations (GEE) were used to account for repeated measures on HCP participating in multiple influenza seasons. In all adjusted models, we included sex, age, occupation and hospital in the model a priori to be consistent with a prior study in the same cohort [21]. Variables of interest significantly associated with the ARI, influenza-associated ARI and/or RSV-associated ARI in the univariate Poisson regression were included in the multivariable models. We considered p values < 0.05 statistically significant.

Among ARI episodes, rates of presenteeism and absenteeism were calculated as the percentage of ARI episodes associated with presenteeism or absenteeism, and 95% Clopper–Pearson confidence intervals (CI) were estimated [13]. We also calculated cumulative hours of missed work among all ARI episodes by summing the total number of scheduled work hours that participants missed during a period when they were sick with an ARI. The HCP were considered unable to do usual activities if they rated their ability to perform daily activities as 0–6, and able to do usual activities if they rated their ability to perform daily activities as 7–9. Like above, factors associated with presenteeism- and absenteeism-related ARI episodes were assessed by univariate and multivariable logistic GEE.

We used multiple imputation by chained equations [22] to impute laboratory results for ARI without respiratory samples and to impute presenteeism and absenteeism from ARI without such information. The imputation models included the same variables as those in the analytic models and auxiliary variables that are either associated with the missing mechanism or correlated with the missing data. Twenty imputed datasets were generated, and parameter estimates from each dataset were pooled to obtain summary metrics for analyses. Finally, we conducted sensitivity analyses that used person-time as the denominator when calculating relative rates to examine how accounting for person-time accrual might affect the findings. An HCP accrued person-time at risk during weeks when influenza virus was detectable in the cohort across three influenza seasons. The duration of ARI and a 14-day post-ARI refractory period were subtracted from person-time calculations overall for any ARI and separately by viral aetiology. We used Poisson GEE with person-time as an offset term to calculate relative rate. We also conducted sensitivity analyses including both complete case analyses and an additional analysis of risk factors for presenteeism and absenteeism, with influenza vaccination status forcibly included in the model. All analyses were conducted with SAS software, version 9.4 (SAS Institute).

## Results

### Demographical data

**Figure 1:**
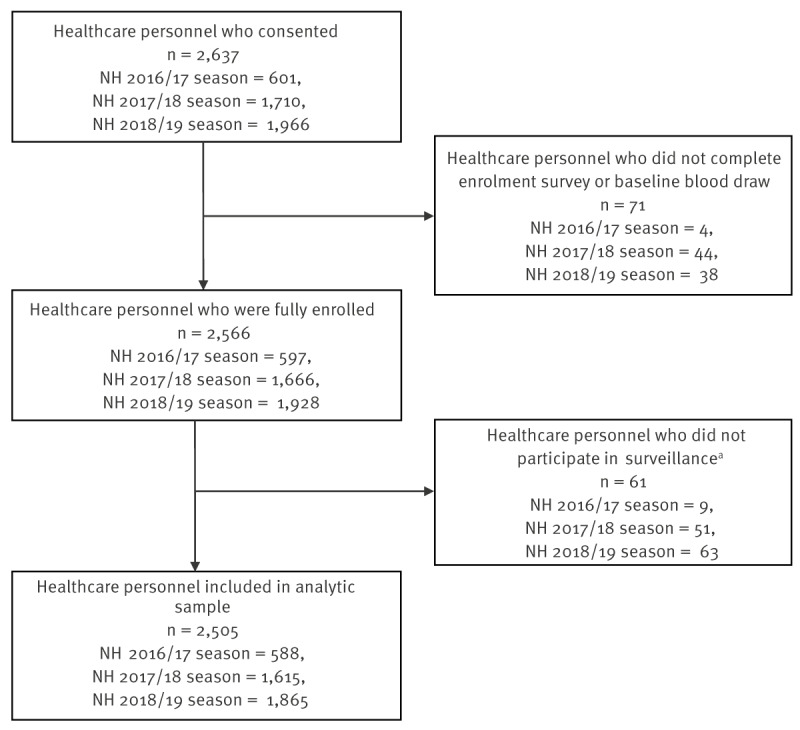
Flowchart for recruitment of participants, study on respiratory infection and presenteeism/absenteeism among healthcare personnel, Israel, 2016–2019 influenza seasons (n = 2,637)

From 2016 to 2019, we obtained informed consent and enrolled a total of 2,637 HCP (Figure 1). 

NH: northern hemisphere.

^a^ Participants were excluded if they enrolled in the study but could not be reached (“missed surveillance”) in any week of the influenza season.

Some participants were involved in multiple years. The totals per box refer to unique individuals involved in at least one of the three seasons.

After excluding 132 (5.0%) HCP without baseline data or surveillance data, 2,505 HCP remained in the analytic dataset completing active surveillance for a median of 100% (interquartile range (IQR): 87–100) of influenza season weeks; 1,242 (49.6%) HCP participated during a single influenza season, 963 (38.4%) in two seasons and 300 (12.0%) in all three seasons. The median age of the HCP at enrolment was 41 years (IQR: 33–51), 70.1% self-identified as female, 86.5% as non-ultra-orthodox Jewish, 11.5% as Arab, 2.0% as ultra-orthodox Jewish, and 50.3% as being of high socioeconomic status (Table 1). Approximately one in four (n = 647; 25.8%) HCP had one or more pre-existing medical conditions at enrolment documented by electronic medical records. Most HCP were nurses (n = 1,426; 56.9%), followed by physicians (n = 630; 25.1%) and allied personnel (e.g. medical assistants and support personnel) (n = 449; 17.9%). Less than half (48.7%) of the HCP had received the current season’s influenza vaccine during the enrolment year.

**Table 1:** Demographic characteristics among healthcare personnel cohorts, Israel, 2016–2019 influenza seasons (n = 2,505)

	Unique participants^a^ n = 2,505		Year of enrolment						p value^b^		
			2016 n = 588		2017 n = 1,615		2018 n = 1,865		ARI	Influenza	RSV
	n	Col%	n	Col%	n	Col%	n	Col%			
**Demographic characteristics**											
**Hospital**											
Soroka Medical Center	1,286	51.3	275	46.8	772	47.8	984	52.8	**< 0.0001**	**< 0.0001**	0.88
Rabin Medical Center	1,219	48.7	313	53.2	843	52.2	881	47.2			
**Sex (binary variable)**											
Male	750	29.9	173	29.4	487	30.2	526	28.2	0.06	**0.05**	0.42
Female	1,755	70.1	415	70.6	1,128	69.8	1,339	71.8			
**Age in years**											
18–34	744	29.7	163	27.7	439	27.2	461	24.7	**< 0.0001**	0.63	0.52
35–49	1,079	43.1	275	46.8	724	44.8	832	44.6			
≥ 50	682	27.2	150	25.5	452	28.0	572	30.7			
**Ethnicity**											
Non-ultra-orthodox Jewish	2,167	86.5	522	88.8	1,407	87.1	1,635	87.7	**0.04**	0.68	0.05
Ultra-orthodox Jewish	49	2.0	13	2.2	34	2.1	36	1.9			
Arab	289	11.5	53	9.0	174	10.8	194	10.4			
**Social economic status [** 40 **]**											
Low	373	14.9	76	12.9	220	13.6	261	14.0	0.80	0.20	0.27
Middle	872	34.8	185	31.5	594	36.8	668	35.8			
High	1,260	50.3	327	55.6	801	49.6	936	50.2			
**Number of children in the household^c^**											
0	1,447	57.8	312	53.1	932	57.8	1,093	58.6	0.42	0.50	0.09
1	424	16.9	107	18.2	273	16.9	302	16.2			
2	385	15.4	102	17.3	245	15.2	285	15.3			
≥ 3	249	9.9	67	11.4	163	10.1	185	9.9			
**Number of bedrooms in the home**											
1	123	4.9	27	4.6	81	5.0	80	4.3	0.27	0.28	0.45
2	397	15.8	82	13.9	257	15.9	285	15.3			
3	783	31.3	193	32.8	511	31.6	554	29.7			
4	620	24.8	147	25.0	398	24.6	480	25.7			
≥ 5	582	23.2	139	23.6	368	22.8	466	25.0			
**Subjective health^d^**											
Excellent	807	32.2	186	31.6	523	32.4	517	27.7	**0.00**	0.91	0.55
Very good	1,030	41.1	232	39.5	648	40.1	807	43.3			
Good	524	20.9	141	24.0	325	20.1	426	22.8			
Fair or poor	144	5.7	29	4.9	119	7.4	115	6.2			
**Index season vaccination^e^**											
Unvaccinated	1,285	51.3	311	52.9	870	53.9	994	53.3	**< 0.0001**	0.96	0.44
Vaccinated	1,220	48.7	277	47.1	745	46.1	871	46.7			
**Occupation characteristics**											
**Occupation**											
Physicians	630	25.1	171	29.1	375	23.2	406	21.8	0.99	0.94	0.84
Nurses, technicians	1,426	56.9	318	54.1	956	59.2	1,105	59.2			
Medical assistants	449	17.9	99	16.8	284	17.6	354	19.0			
**Department regularly worked in**											
**Emergency department**											
No	2,239	89.4	525	89.3	1,448	89.7	1,668	89.4	0.71	0.87	0.33
Yes	266	10.6	63	10.7	167	10.3	197	10.6			
**Intensive care**											
No	2,209	88.2	525	89.3	1,405	87.0	1,642	88.0	0.94	0.11	0.15
Yes	296	11.8	63	10.7	210	13.0	223	12.0			
**Internal medicine**											
No	2,042	81.5	492	83.7	1,312	81.2	1,529	82.0	0.46	0.33	0.46
Yes	463	18.5	96	16.3	303	18.8	336	18.0			
**Paediatrics**											
No	2,278	90.9	513	87.2	1,472	91.1	1,702	91.3	0.32	0.52	0.86
Yes	227	9.1	75	12.8	143	8.9	163	8.7			
**Surgery**											
No	2,088	83.4	492	83.7	1,340	83.0	1,555	83.4	0.39	0.23	0.51
Yes	417	16.6	96	16.3	275	17.0	310	16.6			
**Number of regularly performed aerosol-generating procedures (on average per shift)^f^**											
None	1,185	47.3	287	48.8	715	44.3	912	48.9	0.39	0.71	0.56
1–5	776	31.0	188	32.0	524	32.4	524	28.1			
6–12	544	21.7	113	19.2	376	23.3	429	23.0			
**Hours spent in face-to-face contact with patients**											
≤ 10 h per week	509	20.3	147	25.0	313	19.4	309	16.6	0.21	0.85	0.52
11–30 h per week	526	21.0	125	21.3	355	22.0	369	19.8			
31–50 h per week	1,470	58.7	316	53.7	947	58.6	1,187	63.6			
**Medical conditions**											
**Immunocompromised**											
No	2,213	88.3	517	87.9	1,549	95.9	1,652	88.6	0.45	0.39	0.27
Yes	292	11.7	71	12.1	66	4.1	213	11.4			
**Chronic pulmonary disease**											
No	2,443	97.5	581	98.8	1,585	98.1	1,824	97.8	0.10	**0.005**	0.51
Yes	62	2.5	7	1.2	30	1.9	41	2.2			
**Haematologic disorder**											
No	2,423	96.7	575	97.8	1,578	97.7	1,825	97.9	0.14	0.09	0.08
Yes	82	3.3	13	2.2	37	2.3	40	2.1			
**Chronic cardiovascular disease**											
No	2,467	98.5	583	99.1	1,594	98.7	1,842	98.8	0.21	0.09	0.14
Yes	38	1.5	5	0.9	21	1.3	23	1.2			
**Cancer**											
No	2,368	94.5	546	92.9	1,526	94.5	1,760	94.4	0.21	0.75	0.47
Yes	137	5.5	42	7.1	89	5.5	105	5.6			
**Gastrointestinal disease**											
No	2,421	96.7	573	97.4	1,581	97.9	1,817	97.4	0.38	0.70	0.66
Yes	84	3.3	15	2.6	34	2.1	48	2.6			
**Metabolic disorder**											
No	2,287	91.3	550	93.5	1,522	94.2	1,725	92.5	0.09	0.06	0.06
Yes	218	8.7	38	6.5	93	5.8	140	7.5			
**Neuromuscular disease**											
No	2,435	97.2	571	97.1	1,588	98.3	1,820	97.6	0.84	0.96	0.92
Yes	70	2.8	17	2.9	27	1.7	45	2.4			

ARI: acute respiratory illness; Col%: column %; HCP: healthcare personnel; RSV: respiratory syncytial virus.

^a^ 1,242 HCP participated in one season only, 963 participated in two seasons, and 300 participated in all three seasons.

^b^ p value was calculated using univariate Poisson regression with robust sandwich variance estimator.

^c^ Two HCP did not report the number of children in their household in 2017.

^d^ At the beginning of each season, participants self-reported their overall health status.

^e^ Healthcare personnel were considered vaccinated if they had received an influenza vaccine before the peak of the influenza season.

^f^ Aerosol-generating procedures included collecting respiratory specimens or sputum, administering nebulisers, applying supplemental O_2_ through nasal canula or face masks, applying mechanical ventilation, performing intubations, suction, chest physiotherapy, bedside bronchoscopy, or manual ventilation, and inserting nasogastric tubes.

Entries in bold indicate statistical significance.

### Clinical presentation and laboratory results of acute respiratory infections

**Figure 2:**
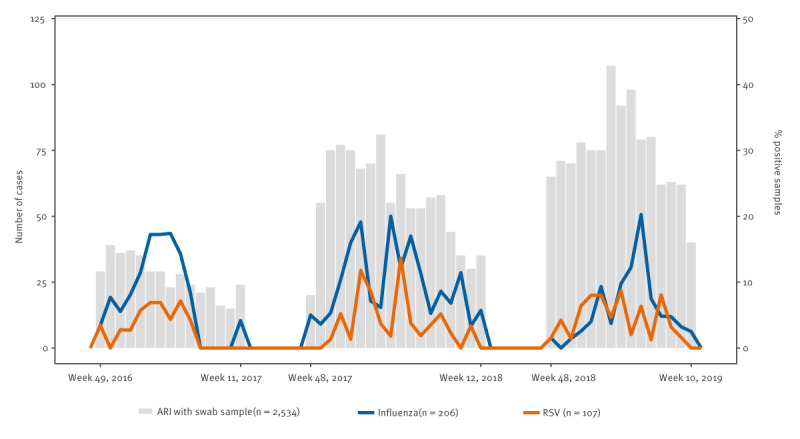
Healthcare personnel with laboratory-confirmed influenza and respiratory syncytial virus-associated acute respiratory infections and percentage of positive samples, by epidemic week, Israel, 2016–2019 influenza seasons (n = 2,534 ARI episodes with swabs)

ARI: acute respiratory infection; RSV: respiratory syncytial virus.

In total, HCP developed 3,202 ARI episodes (cough, feverishness, rhinorrhoea or body aches) between 2016 and 2019 and had a median of one ARI episode per person-season (IQR: 0–1) (Table 2, Figure 2). The HCP provided respiratory swabs during 2,534 (79%) episodes of ARI and reported data on presenteeism and absenteeism in 2,655 (83%) episodes of ARI. The most common reason for not providing a swab was that participants could not self-collect a swab within 7 days of illness onset. The mean time from symptom onset to swab collection was 4 days (IQR: 2–5). Of the 2,534 ARI episodes with specimens collected, 206 (8%) tested positive for influenza and 107 (4%) tested positive for RSV by RT-PCR. Of the influenza-positive specimens, 34 were collected during the 2016/17 season, all of which were A(H3N2) infections, 102 were collected during the 2017/18 season, of which 72% were influenza B/Yamagata and 28% were influenza A viruses (A(H1N1)pdm09 and A(H3N2) subtypes), and 70 specimens were collected during the 2018/19 season, of which 66% were influenza A(H3N2), 31% were A(H1N1)pdm09 and 3% were unsubtyped influenza A virus. Less than 2% of 313 influenza- or RSV-positive specimens were codetections (one (0.3%) influenza A(H3) and A(H1)), four (1%) influenza and RSV).

**Table 2:** Respiratory illnesses among healthcare personnel cohorts, Israel, 2016–2019 influenza seasons (n = 2,505)

	Total	Influenza season		
		2016/17	2017/18	2018/19
ARI	3,202	472	1,328	1,402
Median duration of ARI in days (IQR)	9 (6–12)	8 (5–11)	9 (6–13)	9 (6–12)
Influenza	206	34	102	70
Median duration of influenza in days (IQR)	9 (7–14)	8 (6–12)	10 (8–15)	10 (7–15)
RSV	107	13	43	51
Median duration of RSV in days (IQR)	11 (8–17)	9 (7–11)	13 (9–17)	10 (6–17)

ARI: acute respiratory illness; IQR: interquartile range; RSV: respiratory syncytial virus.

Among 947 unvaccinated HCP who contributed pre- and post-season sera and never tested positive by RT-PCR for influenza virus during follow-up, 177 (19%) seroconverted during the influenza seasons between 2016 and 2019 (51 of 281 (18%) in 2016/17, 31 of 132 (23%) 2017/18, and 95 of 534 (18%) during 2018/19). Of the 177 HCP who seroconverted, less than half (n = 77; 44%) had an episode of ARI with a corresponding influenza-negative swab, and 27 (15%) had an ARI episode with no swab collected; the remaining 73 (41%) did not report an ARI.

### Incidence and factors associated with acute respiratory infection

The adjusted incidence of ARI episodes among HCP was 78.7 per 100 person-seasons (95% CI: 66.5–93.2); 79.5 per 100 person-seasons (95% CI: 65.9–95.9) during 2016/17, 83.4 per 100 person-seasons (95% CI: 70.3–98.8) during 2017/18, and 73.6 per 100 person-seasons (95% CI: 62.0–87.2) during 2018/19 (Table 3). The HCP ≥ 35 years had a lower adjusted relative rate (aRR) of ARI than younger HCP (i.e. aged 35–49 years: aRR = 0.80 (95% CI: 0.73–0.88) and aged ≥ 50 years: aRR = 0.74; 95% CI: 0.67–0.82). The aRR of ARI was higher among non-ultra-orthodox Jewish HCP compared with Arab HCP (aRR = 1.25; 95% CI 1.06–1.47); the risk ARI did not differ by HCP’s sex or occupation. Those HCP who reported fair or poor health status had a higher aRR of ARI compared with those reporting excellent health status (aRR = 1.38; 95% CI: 1.16–1.63).

**Table 3:** Cumulative incidence and relative rate of acute respiratory illness and RT-PCR-confirmed influenza or respiratory syncytial virus infection among healthcare personnel by sociodemographic and occupational factors, Israel, 2016–2019 influenza seasons (n = 2,505)

Variables	ARI				Influenza				RSV			
	Cumulative incidence	95% CI^a^	aRR	95% CI^a^	Cumulative incidence	95% CI^a^	aRR	95% CI^a^	Cumulative incidence	95% CI^a^	aRR	95% CI^a^
Overall	78.7	66.5–93.2	NA		9.1	5.8–14.2	NA		2.5	0.9–7.1	NA	
Season												
NH 2016/17	79.5	65.9–95.9	Reference		9.4	5.5–16.1	Reference		1.9	0.6–6	Reference	
NH 2017/18	83.4	70.3–98.8	1.05	0.95–1.15	12.8	7.9–20.6	**1.36**	**1.12–1.65**	2.6	0.9–7.7	1.34	0.98–1.84
NH 2018/19	73.6	62–87.2	0.92	0.84–1.02	6.2	3.8–10.1	**0.66**	**0.55–0.79**	3.1	1.1–9.3	**1.62**	**1.23–2.14**
Hospital												
Soroka Medical Center	89.6	75.1–106.9	Reference		12.4	7.8–19.8	Reference		2.5	0.9–7.4	Reference	
Rabin Medical Center	69.1	58.3–82	**0.77**	**0.71–0.83**	6.6	4.1–10.8	**0.53**	**0.45–0.63**	2.5	0.9–7.2	0.97	0.77–1.22
Sex (binary variable)												
Male	75.4	63–90.2	Reference		7.5	4.4–12.9	Reference		2.9	1–8.8	Reference	
Female	82.2	69.2–97.6	1.09	0.99–1.2	10.9	6.9–17.2	**1.44**	**1.14–1.82**	2.2	0.7–6.3	**0.74**	**0.57–0.98**
Age in years												
18–34	93.8	78.5–112.1	Reference		10.3	6.1–17.5	Reference		3.3	1.1–9.8	Reference	
35–49	74.8	62.7–89.3	**0.80**	**0.73–0.88**	8.6	5.4–13.9	0.84	0.67–1.05	2.5	0.9–7.3	0.76	0.56–1.03
≥ 50	69.5	58.1–83.1	**0.74**	**0.67–0.82**	8.3	5–14.1	0.81	0.64–1.03	1.9	0.6–5.9	**0.56**	**0.39–0.81**
Occupation												
Physicians	80.2	67.1–96	Reference		10.0	5.8–17.1	Reference		2.7	0.9–8.7	Reference	
Nurses, technicians	77.2	65.1–91.6	0.96	0.87–1.06	9.1	5.4–15.3	0.91	0.74–1.11	2.6	0.9–7.4	0.95	0.71–1.29
Medical assistants	78.8	64.9–95.6	0.98	0.86–1.12	8.2	4.8–13.9	0.82	0.64–1.06	2.2	0.7–7	0.81	0.57–1.15
Ethnicity												
Non-ultra-orthodox Jewish	89.4	78.1–102.2	**1.25**	**1.06–1.47**	8.5	6.1–11.9	1.25	0.91–1.71	3.2	1.3–7.8	**3.42**	**1.58–7.44**
Ultra-orthodox Jewish	76.3	57.4–101.4	1.07	0.79–1.43	12.8	4.9–33.2	**1.88**	**1.07–3.32**	5.2	1–28	**5.50**	**1.93–15.66**
Arab	71.6	57.8–88.6	Reference		6.8	3.6–12.8	Reference		0.9	0.3–3.5	Reference	
Influenza vaccination status												
Unvaccinated	70.8	59.6–84.2	Reference		9.1	5.7–14.5	Reference		2.7	0.9–8.2	Reference	
Vaccinated	87.5	73.5–104	**1.23**	**1.15–1.33**	9.1	5.6–14.7	1.00	0.84–1.18	2.3	0.8–6.5	0.83	0.67–1.04
Subjective health												
Excellent	68.2	57.1–81.5	Reference		8.0	4.8–13.4	Reference		1.8	0.6–5.6	Reference	
Very good	72.3	60.7–86.1	1.06	0.97–1.16	9.8	6.1–16	**1.23**	**1.02–1.47**	2.1	0.7–6.2	1.17	0.88–1.57
Good	83.0	69.4–99.2	**1.22**	**1.09–1.36**	8.9	5.3–15.2	1.12	0.88–1.41	2.6	0.9–8	**1.47**	**1.06–2.03**
Fair or poor	93.8	75.3–116.9	**1.38**	**1.16–1.63**	9.6	4.9–18.6	1.19	0.84–1.69	4.0	1.2–12.6	**2.21**	**1.51–3.23**
Chronic pulmonary disease												
No	73.9	66.3–82.4	Reference		5.5	3.8–8.1	Reference		3.1	1.6–5.7	Reference	
Yes	83.9	63.2–111.2	1.13	0.87–1.47	14.9	7.7–28.9	**2.71**	**2–3.67**	2.1	0.3–12.4	0.67	0.24–1.85

ARI: acute respiratory infection; CI: confidence interval; NA: not applicable; HCP: healthcare personnel; NH: northern hemisphere; aRR: adjusted relative rate; RSV: respiratory syncytial virus.

^a^ Cumulative incidence and RR were estimated using multivariable Poisson regression with robust sandwich variance estimator.

Entries in bold indicate statistical significance.

### Incidence and factors associated with acute respiratory infection by viral aetiology

The adjusted incidence of RT-PCR-confirmed influenza ARI was 9.1 per 100 person-seasons (95% CI: 5.8–14.2) and similar among vaccinated and unvaccinated HCP (Table 3). Incidence of influenza-associated ARI was highest in 2017/18 (12.8 per 100 person-seasons; 95% CI: 7.9–20.6), when influenza B/Yamagata was the predominant lineage (72% of detections) and lowest in 2018/19 (6.2 per 100 person-seasons; 95% CI: 3.8–10.1) when influenza A(H3N2) was the predominant subtype (66% of detections) (Table 3). Influenza-associated ARI lasted a median duration of 9 days (IQR: 7–14). The aRR of influenza-associated ARIs was higher among female compared with male HCP (aRR = 1.44; 95% CI: 1.14–1.82) and among HCP with chronic pulmonary conditions compared with those without (aRR = 2.71; 95% CI: 2.00–3.67); the aRR of influenza-associated ARIs was similar across age groups. Among unvaccinated HCP, the incidence of RT-PCR-confirmed and laboratory-confirmed (RT-PCR-confirmed or seroconversion) influenza illness was 9.1 per 100 person-seasons (95% CI: 5.7–14.5) and 27.5 per 100 person-seasons (95% CI: 17.8–42.5), respectively.

The adjusted incidence of RT-PCR-confirmed RSV ARI was 2.5 per 100 person-seasons (95% CI: 0.9–7.1); 1.9 per 100 person-seasons (95% CI: 0.6–6.0) in 2016/17, 2.6 per 100 person-seasons (95% CI: 0.9–7.7) in 2017/18, and 3.1 per 100 person-seasons (95% CI: 1.1–9.3) in 2018/19 (Table 3). The RSV-associated ARI lasted a median duration of 11 days (IQR: 8–17). Healthcare personnel aged ≥ 50 years had a lower aRR of RSV-associated ARI than HCP aged 18–34 years (aRR = 0.56; 95% CI: 0.39–0.81). Those HCP who reported fair or poor health status had a higher aRR of RSV-associated ARI compared with those reporting excellent health status (aRR = 2.21; 95% CI: 1.51–3.23).

When we used person-time as a denominator in sensitivity analyses, findings on relative rates of ARI and influenza- or RSV-associated ARI were similar. Complete case analyses indicated consistent point estimates with wider confidence intervals because of the smaller analytic sample. Including influenza vaccination status in the model assessing risk factors for presenteeism and absenteeism did not significantly affect the estimates. The results of these sensitivity analyses are available in the supplementary material.

### Workplace presenteeism and absenteeism

During the influenza seasons from 2016 to 2019, HCP worked for at least 1 day despite being ill (presenteeism) during 92% (95% CI: 91–93) of all ARI episodes (Table 4). The incidence of ARI-related presenteeism over the 3-year study period was 72.3 per 100 person-seasons (95% CI: 60.4–86.4). Decreased job performance was reported during 823 (36%) ARI episodes with presenteeism. In a multivariable model, HCP aged ≥ 50 years were 60% more likely to present to work while ill compared with those aged 18–34 years (aOR = 1.60; 95% CI: 1.07–2.39) (Table 5). Presenteeism during ARI was 88% more common among medical assistants than physicians (aOR = 1.88; 95% CI: 1.02–3.48). Those HCP working in surgery department were 60% more likely to present to work while ill compared with those who did not (aOR = 1.60; 95% CI: 1.02–2.52). Among HCP with influenza-associated ARIs, 92% (95% CI: 87–96) worked for at least 1 day despite being ill, with an incidence of 7.4 per 100 person-seasons (95% CI: 4.2–12.9) (Table 4). Similarly, presenteeism was reported during 97% (95% CI: 91–99) of RSV-associated ARI episodes, with an incidence of 2.2 per 100 person-seasons (95% CI: 0.8–6.4).

**Table 4:** Presenteeism and absenteeism rates and cumulative incidence of acute respiratory infections per 100 person-seasons, Israel, 2016–2019 influenza seasons (n = 2,505)

Outcome	Point estimate^a^	95% CI
ARI		
Presenteeism rate	92.2%	91.2–93.2
Incidence of ARI-associated presenteeism	72.3	60.4–86.4
Absenteeism rate	38.0%	36.1–39.8
Incidence of ARI-associated absenteeism	36.3	29.1–45.4
RT-PCR-confirmed influenza		
Presenteeism rate	92.0%	86.9–95.5
Cumulative incidence of influenza-associated presenteeism	7.4	4.2–12.9
Absenteeism rate	59.8%	52.1–67.1
Cumulative incidence of influenza-associated absenteeism	4.8	2.6–8.8
RT-PCR-confirmed RSV		
Presenteeism rate	96.7%	90.6–99.3
Cumulative incidence of RSV-associated presenteeism	2.2	0.8–6.4
Absenteeism rate	32.2%	22.8–42.9
Cumulative incidence of RSV-associated absenteeism	1.4	0.4–4.6

ARI: acute respiratory illness; CI: confidence interval; RSV: respiratory syncytial virus.

^a^ For the presenteeism/absenteeism rate, 95% Clopper–Pearson confidence intervals were calculated. Cumulative incidence was estimated using multivariable Poisson regression with robust sandwich variance estimator.

**Table 5:** Risk factors for acute respiratory illness associated presenteeism and absenteeism among healthcare personnel, Israel, 2016–2019 influenza seasons (n = 3,202 episodes)

Variables	Presenteeism		Absenteeism	
	aOR^a^	95% CI	aOR^a^	95% CI
Hospital				
Soroka Medical Center	Reference		Reference	
Rabin Medical Center	1.00	0.73–1.37	1.06	0.90–1.25
Sex (binary variable)				
Male	Reference		Reference	
Female	1.11	0.78–1.57	**1.25**	**1.02–1.52**
Age in years				
18–34	Reference		Reference	
35–49	1.21	0.88–1.66	0.86	0.71–1.04
≥ 50	**1.60**	**1.07–2.39**	1.15	0.93–1.42
Occupation				
Physicians	Reference		Reference	
Nurses, technicians	1.26	0.87–1.82	1.10	0.89–1.36
Medical assistants	**1.88**	**1.02–3.48**	1.19	0.91–1.55
Subjective health				
Excellent	Reference		Reference	
Very good	1.12	0.77–1.62	1.11	0.91–1.35
Good	0.69	0.46–1.03	**1.30**	**1.05–1.62**
Fair or poor	0.84	0.45–1.60	1.27	0.90–1.79
Regularly worked in surgery department				
No	Reference		Reference	
Yes	**1.60**	**1.02–2.52**	0.85	0.69–1.06
Number of performed aerosol-generating procedures (on average per shift)				
None	Reference		Reference	
1–5	0.80	0.56–1.15	0.83	0.68–1.02
6–12	0.72	0.49–1.07	**0.65**	**0.52–0.82**
Chronic pulmonary disease				
No	Reference		Reference	
Yes	1.06	0.43–2.66	**1.69**	**1.03–2.77**

CI: confidence interval; aOR: adjusted odds ratio.

^a^ Odds ratios were estimated using multivariable logistic regression with robust sandwich variance estimator to account for healthcare personnel reporting more than one illness episode.

Entries in bold indicate statistical significance.

Being absent from work for at least one shift while ill (absenteeism) occurred in 38% (95% CI: 36–40) of ARI episodes, with an incidence of 36.3 per 100 person-seasons (95% CI: 29.1–45.4). Each season, among HCP who were absent from work at least once during an episode of ARI, participants missed work for a median of 16 h (IQR: 8–24) per season because of illness with ARI; during the influenza seasons from 2016 to 2019, ill HCP missed 23,308 cumulative hours (95% CI: 21,985–24,631). In a multivariable model, absenteeism during ARI was 25% higher among female compared with male HCP (aOR = 1.25; 95% CI: 1.02–1.52) and 30% higher among HCP who reported good health status compared with those reporting excellent health status (aOR = 1.30; 95% CI: 1.05–1.62) (Table 5). Those HCP with a chronic pulmonary condition were at higher odds of absenteeism compared with those without (aOR = 1.69; 95% CI: 1.03–2.77), whereas HCP performing 6–12 aerosolising procedures per shift were 35% less likely to be absent from work while ill compared with those who do not perform such procedures (aOR = 0.65; 95% CI: 0.52–0.82).

Among HCP experiencing influenza-associated ARIs, absenteeism was reported in 60% (95% CI: 52–67) of the episodes. The incidence of absenteeism during an influenza-associated ARI was 4.8 per 100 person-seasons (95% CI: 2.6–8.8). The HCP missed work because of an influenza-associated ARI for a median duration of 16 h (IQR: 8–32) or 3,053 cumulative hours (95% CI: 2,541–3,566). Absenteeism was reported in 32% (95% CI: 23–43) of RSV-associated ARI. The incidence of absenteeism during an RSV-associated ARI episode was 1.4 per 100 person-seasons (95% CI: 0.4–4.6), and HCP missed work for a median duration of 16 h (IQR: 8–24) or 876 cumulative hours (95% CI: 578–1,173).

## Discussion

Influenza- and RSV-associated ARI were common among HCP in two large Israeli hospitals and frequently associated with presenteeism and absenteeism; these findings provide additional evidence of the potential value of measures to decrease respiratory infections among HCP, such as improving influenza vaccination coverage, optimising infection prevention and control and encouraging sick HCP to stay home to reduce the incidence of ARI and thereby decrease the risk of in-hospital transmission of viruses to staff and patients. While the incidence of RT-PCR-confirmed influenza illness among unvaccinated HCP was 9.1 per 100 person-seasons, the incidence more than doubled when combined with serological evidence of infection. These high rates of influenza among unvaccinated HCP are consistent with results from a recent systematic review, where the incidence of influenza illness and influenza infection among HCP was 7.5 (95% CI: 4.9–11.7) and 18.7 (95% CI: 15.8–22.1), respectively [2].

Although influenza viruses were the most frequently detected viruses, RSV infection also caused many ARI episodes among HCP. The risk of RSV we observed was consistent with results among HCP working in a hospital paediatrics department in California where approximately one in 20 HCP developed RSV illnesses: 5% among HCP who used personal protective equipment and 61% among HCP who did not [23]. While RSV vaccines are approved in the European Union for pregnant women and adults aged > 60 years and in Israel for adults aged > 60 years [24], our findings of high RSV incidence, combined with the occupational risk while caring for ill patients [23,25], suggest that HCP may also be an important population to immunise against RSV with currently licensed products or others in development [26,27].

We found presenteeism was very common; HCP worked at least 1 day while ill during nine of 10 ARI episodes. A previous study of HCP in this same cohort found that high rates of presenteeism may be related to a strong personal work ethic and an institutional culture that discourages taking sick leave [3]. High rates of presenteeism among HCP have also been described elsewhere [28,29]. During the influenza season, 94.6% of HCP in Canada worked at least 1 day while ill with ARI, and this happened more frequently in the absence of paid sick leave [23]. In the TransFLUas influenza transmission study, 67.9% of HCP with laboratory-confirmed influenza at the University of Zurich worked while ill [30]. While age and provider role have not been identified in other cohorts at our study sites as risk factors for presenteeism, older HCP and those regularly working in the surgery department seemed more reluctant to stay home while ill. Healthcare personnel who worked while ill with influenza also have been shown to infect co-workers and vulnerable patients [29,31]. The risk of transmission is probably highest during the first days of illness, when people are most likely to shed viruses. Persistent ARI symptoms could also impair HCP’s ability to competently perform their duties while ill. The United States Centers for Disease Control and Prevention recommends that HCP stop patient care, don a mask and notify a supervisor when ill [32].

We also found that workplace absenteeism was common during ARI, as more than one in three ARI episodes resulted in missed workdays or > 23,000 h of lost work among our cohort of HCP. An association between ARIs and absenteeism has been observed previously [33]. In Italy, HCP missed > 11,000 working days per year, resulting in an annual cost to society of ca EUR 1.7 million because of influenza illnesses [34]. A healthy workforce helps ensure that healthcare systems are resilient. The work absences among HCP in Italy occurred during annual influenza epidemics, periods of time when healthcare systems need staff for surge capacity. Such potential erosion in the pool of healthy HCP can be even more problematic during a pandemic.

While a recent systematic literature review found that HCP who received an influenza vaccine had on average ca 64% (RR = 0.36; 95% CI: 0.25–0.54) lower risk of influenza virus infection and influenza-associated absenteeism compared with unvaccinated HCP [35], vaccines may be less effective in preventing mild illness during certain epidemics and among persons repeatedly exposed to influenza antigens. There may also be bias by indication, where persons who self-identify as being at higher risk of ARI get vaccinated more frequently than others, hoping to protect themselves against influenza. In our cohort, standard dose influenza vaccines were not effective during the period 2016 to 2019 at preventing influenza ARI among HCP at Soroka and Rabin Medical Center [21]. However, new enhanced influenza vaccines that have recently become more widely available may offer better protection: Recent clinical trials suggest that cell-based influenza vaccines may provide superior immunogenicity among HCP, compared with egg-based influenza vaccines [36]; other clinical trials are ongoing among HCP to explore whether high-dose or adjuvanted influenza vaccines could provide superior protection to HCP [37].

Infection control practices such as vaccination, frequent handwashing, disinfection of equipment and the proper use of personal protective equipment can prevent nosocomial infections [32]. Less than half of the Soroka and Rabin Medical Center HCP received the influenza vaccine each season. Additional efforts at healthcare facilities to increase influenza vaccination uptake among HCP could be undertaken to address these low coverage rates. Sick HCP should also stay at home when they are sick to reduce the risk of infecting colleagues and vulnerable patients [33]. Previous research in Israel has identified several reasons why HCP choose to work while sick, including the belief that influenza is not serious enough to prevent working [3]. In addition to efforts aimed at increasing vaccination uptake, hospitals could offer paid sick leave to discourage presenteeism or explore the cost-effectiveness of antiviral treatment within 48 h of illness onset to decrease illness duration and the risk of complications [38].

Our study had notable strengths. We prospectively followed HCP twice a week in two hospitals during three seasons with relatively high active surveillance completion, and nearly four of five symptomatic HCP self-collected their swabs. Self-collection has been shown to be a reliable diagnostic method for influenza with relatively high sensitivity and specificity [16]. While we relied on RT-PCR-confirmed influenza-like illness to quantify influenza incidence in previous cohorts [20], we used a sensitive case definition of acute respiratory infection and obtained pre- and post-season serology to improve the probability of identifying asymptomatic infection. Data on presenteeism and absenteeism were identified from a combination of illness surveys and data from hospital human resources departments, making our findings more robust.

Our study had limitations. Fewer HCP were enrolled in the 2016/17 influenza season than in the other two seasons, resulting in fewer swabs tested that year. However, the percentage of participants who reported ARI and had swabs collected was similar throughout all three seasons. The study occurred before the COVID-19 pandemic and HCP attitudes about working while sick may have changed since the pandemic. While our findings about presenteeism and absenteeism present robust pre-pandemic data, rates of presenteeism and absenteeism should be re-evaluated because of potentially altered attitudes and policies, for example, about masking and working while sick. Influenza-associated ARI episodes may have been missed when swabs were not collected or collected too late [15]. However, we used multiple imputation to address the potential underestimation of influenza incidence because of missed detections. Incidence of RSV-associated ARI may not fully reflect RSV incidence and risk among HCP in our study because active surveillance was only conducted during the influenza season which can occur after peak RSV circulation in Israel [11]. While we used a sensitive ARI case definition, some pauci-symptomatic illnesses among persons infected with influenza may have been missed [20]. If influenza infections occurred early in the season, it is possible that they would have been identified as seronegative at the end of the season and incorrectly classified as uninfected if there had been considerable waning of their antibody titres [39].

## Conclusion

We found high incidence of acute respiratory illness and influenza and RSV infections among HCP at two large hospitals in Israel during three influenza seasons. The HCP commonly reported workplace presenteeism and absenteeism. Improving influenza vaccination coverage, considering the introduction of RSV vaccines among HCP, optimising hospital infection prevention and control measures to reduce the burden of ARI (e.g. frequent hand hygiene, use of gloves, gowns, and face masks for patients under droplet precautions, isolation or cohorting of influenza or RSV cases, caution when performing aerosol-generating procedures, environmental surface cleaning etc.), and encouraging sick HCP to stay at home are all important strategies to reduce the incidence of ARI among HCP and decrease the risk of in-hospital transmission of viruses to staff and patients and optimise healthcare system resilience. Further studies are needed to examine strategies to optimise such interventions.

## Supplementary Data

229000 Supplement
