# Supplementary material for: Incidence of laboratory-confirmed influenza and RSV and associated presenteeism and absenteeism among healthcare personnel, Israel, influenza seasons 2016 to 2019
Source: Euro Surveill. 2024 Aug 1;29(31):2300580. doi: 10.2807/1560-7917.ES.2024.29.31.2300580 (PMC11295438; doi:10.2807/1560-7917.ES.2024.29.31.2300580)
Supplement: Supplement [file 23-00580_BAUMGARTNER_Supplement.pdf]

Supplementary materials for

Incidence of laboratory-confirmed influenza and RSV and associated presenteeism and absenteeism among health care personnel in Israel, 2016–2019

Disclaimer: This supplementary material is hosted by *Eurosurveillance* as supporting information alongside the article *Incidence of laboratory-confirmed influenza and RSV and associated presenteeism and absenteeism among health care personnel in Israel, 2016–2019*, on behalf of the authors, who remain responsible for the accuracy and appropriateness of the content. The same standards for ethics, copyright, attributions and permissions as for the article apply. Supplements are not edited by *Eurosurveillance* and the journal is not responsible for the maintenance of any links or email addresses provided therein.

Supplementary Table S1. Relative rate of acute respiratory illness (ARI), RT-PCR confirmed influenza, and RT-PCR confirmed respiratory syncytial virus (RSV) among healthcare personnel (HCP) by sociodemographic and occupational factors during the 2016–2019 influenza seasons, N=2,505 participants

	ARI	Influenza	RSV
**Variables**	**Relative Rate (95% CI) [1]**	**Relative Rate (95% CI) [1]**	**Relative Rate (95% CI) [1]**
Season			
NH 2016/17	REF	REF	REF
NH 2017/18	0.98 (0.87 - 1.10)	**1.28 (1.06 - 1.55)**	1.26 (0.92 - 1.72)
NH 2018/19	0.96 (0.85 - 1.08)	**0.7 (0.58 - 0.83)**	**1.76 (1.33 - 2.33)**
Hospital			
Soroka Medical Center	REF	REF	REF
Rabin Medical Center	**0.73 (0.66 - 0.81)**	**0.53 (0.45 - 0.63)**	1.18 (0.94 - 1.48)
Sex			
Male	REF	REF	REF
Female	1.13 (1.00 - 1.27)	**1.45 (1.15 - 1.83)**	**0.71 (0.54 - 0.93)**
Age			
18-34	REF	REF	REF
35-49	**0.73 (0.65 - 0.82)**	0.83 (0.66 - 1.04)	0.93 (0.69 - 1.26)
≥50	**0.67 (0.59 - 0.77)**	**0.78 (0.61 - 0.99)**	0.7 (0.48 - 1.01)
Occupation			
Physicians	REF	REF	REF
Nurses, technicians	0.93 (0.83 - 1.05)	0.88 (0.72 - 1.07)	0.95 (0.70 - 1.28)
Medical assistants	1.01 (0.85 - 1.19)	0.82 (0.63 - 1.06)	0.83 (0.58 - 1.18)
Ethnicity			
Non-ultra-orthodox Jewish	1.20 (0.98 - 1.47)	1.19 (0.88 - 1.63)	**2.8 (1.30 - 6.02)**
Ultra-orthodox Jewish	1.02 (0.71 - 1.46)	**1.82 (1.03 - 3.20)**	**4.89 (1.74 - 13.74)**
Arab	REF	REF	REF
Influenza Vaccination Status			
Unvaccinated	REF	REF	REF
Vaccinated	**1.15 (1.06 - 1.25)**	1.01 (0.85 - 1.21)	**0.73 (0.58 - 0.91)**
Subjective Health			
Excellent	REF	REF	REF
Very Good	1.08 (0.96 - 1.21)	**1.26 (1.05 - 1.52)**	1.14 (0.86 - 1.53)
Good	**1.31 (1.14 - 1.51)**	1.17 (0.93 - 1.48)	1.28 (0.93 - 1.78)
Fair or Poor	**1.47 (1.17 - 1.83)**	1.24 (0.87 - 1.76)	**1.93 (1.31 - 2.85)**
Chronic Pulmonary			
No	REF	REF	REF
Yes	1.29 (0.95 - 1.75)	**2.86 (2.12 - 3.87)**	0.57 (0.21 - 1.58)

Abbreviation: NH, northern hemisphere

[1] Relative rates were estimated using multivariate generalized estimating equations poisson with log days of follow up as an offset term

Supplementary Table S2. Cumulative incidence and relative risk of RT-PCR-confirmed influenza and RT-PCR-confirmed respiratory syncytial virus (RSV) among healthcare personnel (HCP) by sociodemographic and occupational factors during the 2016–2019 influenza seasons: complete case analyses, N=2,258 participants

Variables	Influenza				RSV			
	Cumulative incidence (95% CI)[1]		Relative risk (95% CI)[1]		Cumulative incidence (95% CI)[1]		Relative risk (95% CI)[1]	
Overall	8.1	(5-13.2)			2.0	(0.6-6.4)		
Season								
NH 2016/17	8.6	(4.9-15.3)	REF		1.7	(0.5-5.7)	REF	
NH 2017/18	11.4	(6.8-19.1)	1.32	(0.89-1.97)	2.0	(0.6-6.7)	1.20	(0.61-2.35)
NH 2018/19	5.4	(3.2-9.1)	**0.62**	**(0.4-0.98)**	2.4	(0.7-7.8)	1.42	(0.73-2.77)
Hospital								
Soroka Medical Center	11.5	(7-19)	REF		2.1	(0.6-6.8)	REF	
Rabin Medical Center	5.7	(3.3-9.7)	**0.49**	**(0.35-0.69)**	1.9	(0.6-6.2)	0.93	(0.58-1.5)
Sex								
Male	6.6	(3.7-11.6)	REF		2.4	(0.7-8.1)	REF	
Female	10.0	(6.1-16.2)	1.52	(0.99-2.34)	1.7	(0.5-5.5)	0.68	(0.34-1.35)
Age								
18-34	9.2	(5.3-16.1)	REF		2.8	(0.9-8.7)	REF	
35-49	7.7	(4.6-12.8)	0.84	(0.57-1.23)	2.1	(0.6-6.5)	0.74	(0.43-1.28)
≥50	7.4	(4.3-12.9)	0.81	(0.52-1.25)	1.4	(0.4-5.3)	0.52	(0.26-1.03)
Occupation								
Physicians	9.0	(5.1-15.9)	REF		2.2	(0.6-7.6)	REF	
Nurses, technicians	7.8	(4.5-13.7)	0.86	(0.57-1.32)	2.0	(0.6-6.6)	0.95	(0.53-1.71)
Medical assistants	7.5	(4.2-13.2)	0.83	(0.47-1.45)	1.8	(0.5-6.4)	0.85	(0.35-2.1)
Ethnicity								
Non-ultra-orthodox Jewish	7.9	(5.5-11.4)	1.35	(0.69-2.63)	2.8	(1-7.6)	**4.19**	**(1.55-11.36)**
Ultra-orthodox Jewish	11.3	(4.1-31.2)	1.92	(0.59-6.24)	4.3	(0.7-27.9)	**6.39**	**(1.04-39.3)**
Arab	5.9	(3-11.7)	REF		0.7	(0.2-2.6)	REF	
Influenza Vaccination Status								
Unvaccinated	8.0	(4.8-13.2)	REF		2.2	(0.7-7.5)	REF	
Vaccinated	8.2	(4.8-13.8)	1.02	(0.74-1.42)	1.8	(0.6-5.7)	0.81	(0.5-1.3)
Subjective Health								
Excellent	7.2	(4.2-12.6)	REF		1.4	(0.4-5.1)	REF	
Very Good	9.0	(5.4-14.9)	1.24	(0.86-1.79)	1.7	(0.5-5.8)	1.20	(0.67-2.15)
Good	8.1	(4.6-14.3)	1.12	(0.7-1.79)	2.2	(0.7-7.2)	1.54	(0.74-3.2)
Fair or Poor	8.1	(4-16.6)	1.12	(0.58-2.17)	3.0	(0.8-10.7)	2.09	(0.94-4.62)
Chronic Pulmonary								
No	5.0	(3.3-7.5)	REF		2.8	(1.4-5.5)	REF	
Yes	13.2	(6.5-26.8)	**2.66**	**(1.41-5.02)**	1.4	(0.2-11.1)	0.51	(0.07-3.74)

Abbreviation: NH, northern hemisphere

[1] Cumulative incidence and relative risk were estimated using multivariable Poisson regression with robust sandwich variance estimator

Supplementary Table S3. Risk factors for acute respiratory illness (ARI) associated presenteeism and absenteeism among healthcare personnel (HCP) during the 2016–2019 influenza seasons: complete case analyses, N=2,655 episodes

	Presenteeism		Absenteeism	
Variables	**aOR[1]**	**(95% CI)**	**aOR[1]**	**(95% CI)**
Hospital				
Soroka Medical Center	REF		REF	
Rabin Medical Center	0.98	(0.71 - 1.35)	1.07	(0.90 - 1.27)
Sex				
Male	REF		REF	
Female	1.10	(0.76 - 1.60)	1.21	(0.99 - 1.49)
Age				
18-34	REF		REF	
35-49	1.20	(0.83 - 1.71)	0.85	(0.69 - 1.04)
≥50	1.53	(0.97 - 2.41)	1.14	(0.90 - 1.44)
Occupation				
Physicians	REF		REF	
Nurses, technicians	1.24	(0.84 - 1.84)	1.11	(0.89 - 1.39)
Medical assistants	**2.00**	**(1.05 - 3.80)**	1.16	(0.87 - 1.56)
Subjective Health				
Excellent	REF		REF	
Very Good	1.15	(0.77 - 1.72)	1.12	(0.91 - 1.37)
Good	0.69	(0.44 - 1.07)	**1.33**	**(1.05 - 1.69)**
Fair or Poor	0.84	(0.44 - 1.62)	1.31	(0.92 - 1.86)
Regularly worked in surgery department				
No	REF		REF	
Yes	**1.59**	**(1.00 - 2.52)**	0.83	(0.65 - 1.06)
Number of performed aerosol procedures				
None	REF		REF	
1 - 5	0.82	(0.55 - 1.23)	0.85	(0.68 - 1.04)
6 - 12	0.74	(0.48 - 1.14)	**0.64**	**(0.50 - 0.80)**
Chronic Pulmonary				
No	REF		REF	
Yes	0.99	(0.40 - 2.47)	**1.69**	**(1.12 - 2.57)**

Abbreviation: aOR, adjusted odds ratio

[1] Odds ratios were estimated using multivariable Poisson regression with robust sandwich variance estimator to account for repeated measurements.

Supplementary Table S4. Risk factors, including influenza vaccination status, for acute respiratory illness (ARI) associated presenteeism and absenteeism among healthcare personnel (HCP) during the 2016–2019 influenza seasons, N=3,202 episodes

	**Presenteeism**		**Absenteeism**	
Variables	**aOR[1]**	**(95% CI)**	**aOR[1]**	**(95% CI)**
Hospital				
Soroka Medical Center	REF		REF	
Rabin Medical Center	0.98	(0.71 - 1.35)	1.07	(0.91 - 1.26)
Sex				
Male	REF		REF	
Female	1.12	(0.79 - 1.58)	**1.24**	**(1.02 - 1.52)**
Age				
18-34	REF		REF	
35-49	1.22	(0.89 - 1.68)	0.86	(0.71 - 1.04)
≥50	**1.59**	**(1.06 - 2.38)**	1.15	(0.94 - 1.42)
Occupation				
Physicians	REF		REF	
Nurses, technicians	1.30	(0.90 - 1.88)	1.09	(0.88 - 1.35)
Medical assistants	**1.97**	**(1.07 - 3.65)**	1.17	(0.89 - 1.53)
Influenza Vaccination Status				
Unvaccinated	REF		REF	
Vaccinated	1.28	(0.95 - 1.71)	0.91	(0.78 - 1.07)
Subjective Health				
Excellent	REF		REF	
Very Good	1.12	(0.77 - 1.62)	1.11	(0.91 - 1.34)
Good	0.69	(0.46 - 1.04)	**1.30**	**(1.04 - 1.62)**
Fair or Poor	0.85	(0.45 - 1.62)	1.26	(0.90 - 1.78)
Regularly worked in surgery department				
No	REF		REF	
Yes	**1.61**	**(1.02 - 2.53)**	0.85	(0.68 - 1.06)
Number of performed aerosol procedures				
None	REF		REF	
1 - 5	0.79	(0.55 - 1.14)	0.83	(0.68 - 1.02)
6 - 12	0.70	(0.47 - 1.05)	**0.66**	**(0.52 - 0.83)**
Chronic Pulmonary				
No	REF		REF	
Yes	1.02	(0.41 - 2.57)	**1.71**	**(1.05 - 2.80)**

Abbreviation: aOR, adjusted odds ratio

[1] Odds ratios were estimated using multivariable Poisson regression with robust sandwich variance estimator to account for repeated measurements.
